# Co-morbidities associated with non-coeliac gluten sensitivity 

**Published:** 2021

**Authors:** Geoffrey Holmes

## Introduction

 In the late 1970s, case reports indicated that some people were sensitive to wheat but did not have coeliac disease (CD) (1-3). This concept was strengthened by a study of nine adult patients attending the General Hospital, Birmingham, UK, around this time with persistent diarrhoea lasting up to 20 years and averaging five years, which was often socially incapacitating and nocturnal, and defied specific diagnosis but responded to a gluten-free diet (GFD) (4). Because there were no specific tests or symptoms overlapping with irritable bowel syndrome (IBS), little curiosity was generated, and matters rested for several years. However, a resurgence of interest has occurred more recently leading to non-coeliac gluten sensitivity (NCGS) being rather grandly called the new frontier of gluten-related disorders (5). Gluten sensitivity is defined as a reaction to gluten in which allergic and autoimmune mechanisms have been excluded (6). Many aspects of this enigmatic condition remain to be explored, but of note, innate immune responses to gluten appear to be involved in its pathogenesis (7), and zonulin has been identified as a possible disease marker (8). Many disorders occur in association with NCGS as also described for coeliac disease (9, 10), and the list is expanding. The associations with NCGS sometimes regarded as non-coeliac wheat sensitivity (NCWS) form the basis of this review.

**Autoimmune diseases**

Over a period of twelve months, 486 patients were identified with NCGS from centres in Italy (11). The diagnosis was highly presumptive and made when patients reported symptoms after gluten ingestion which improved or resolved when gluten was removed from the diet and reappeared when reintroduced. None had a double-blind placebo-controlled trial, which is regarded as the gold-standard for diagnosis and is a limitation of the study. One or more associated autoimmune diseases, as reported by patients on a questionnaire, occurred in 14% of participants and included thyroiditis (the commonest), psoriasis, Grave’s disease, type 1 diabetes mellitus, and Crohn’s disease. The results were presented only as descriptive. 

Another study from Italy investigated the prevalence of autoimmune disorders among a retrospective group of 131 patients and a prospective group of 42 cases with NCWS (12). The diagnosis was based on double-blind placebo-controlled wheat challenge and the absence of small bowel mucosal atrophy. Control groups were 101 individuals with CD and 50 with IBS for the retrospective and 42 and 40 for the prospective parts of the analysis. Those with NCWS showed a frequency of autoimmune disease similar to CD patients but significantly higher than for those with IBS. In the retrospective study, 38/131 (29%) of NCWS cases had an associated disease compared with 21/101 (21%) in CD and 2/50 (4%) in IBS. Similar results were found in the prospective arm of the study. In the retrospective NCWS group, disorders encountered were Hashimoto’s thyroiditis (29 cases), psoriasis (4), type 1 diabetes mellitus (4), mixed connective tissue disease (1), and ankylosing spondylitis (1), much as might be found in CD. Anti-nuclear antibodies were positive in the retrospective study in 46% of NCWS subjects, significantly higher than found in CD (24%) or IBS (2%) patients, a pattern replicated in the prospective group. The presence of HLA-DQ2/DQ8 was significantly higher in the NCWS cases than in controls but significantly lower than in CD and evident in both arms of the study. A family history of CD was found among NCWS patients as also observed in NCGS (11). 

The frequency of autoimmune disorders was determined in 91 patients with NCWS, 76 blood donors, and 55 individuals with IBS (13). Of those with NCWS, 23 (25.3%) had immune diseases, a significantly higher number than in the control groups. Autoimmune thyroiditis occurred in 16 (17.6%) cases and was the most common condition found. Antinuclear antibodies and extractable nuclear antigen were both significantly higher in NCWS patients than in controls. 

**Neuropsychiatric disorders**

The most common neurological disturbance found among a group of 334 patients with NCGS was peripheral neuropathy (54%), followed by cerebellar ataxia (46%) and encephalopathy (10%) (14). These conditions are reminiscent of those that occur in CD, and both are similar with respect to the presence of anti-tissue transglutaminase 6 antibodies. 

Whether gluten might affect mental state in NCGS was addressed in a study of 22 patients with IBS but without CD, whose symptoms were controlled on a GFD. These patients were given three dietary supplement challenges of gluten, whey, and placebo (diet not supplemented) each for 3 days followed by a wash-out period (15). Gluten ingestion induced feelings of depression, but gastrointestinal symptoms were unaffected. In 61 patients with NCGS, gluten-induced depression and a foggy mind were observed, even after only a short exposure time of a few days (16). Sixty patients with depressive illness or bipolar disorder were investigated for the presence of gluten-related disease using a variety of coeliac antibodies, but no associations were uncovered (17). A GFD does not appear to benefit those with autism (18) or psychoses such as schizophrenia (19). However, severe psychosis in a 14-year-old girl was attributed to NCGS and cured by the patient following a GFD (20). Patients with Giles de la Tourette syndrome showed a marked reduction in tics and obsessive-compulsive characteristics after one year on a GFD (21).

**Headaches**

The prevalence of headaches comprising migraine, tension headache cluster headache, hemicrania continua, and trigeminal neuralgia, was determined in 188 patients with CD, 25 with gluten intolerance, 111 with inflammatory bowel disease, and 178 control subjects (22). This was a self-reported investigation, which imposes some limitations on the value of the results. The ID-migraine tool was used to validate the diagnosis. A significantly higher frequency of headache occurred in the three groups of patients compared with controls, the highest percentage (56%) being found in gluten sensitive patients. Migraine, by ID-migraine criteria, occurred significantly more often among those with CD (21%) and gluten sensitivity (40%) than in controls (6%). The prevalence in inflammatory bowel disease (14%) was also significantly higher than for controls. Of interest, eight cases with CD stated that migraines were improved or resolved on a GFD. Decreased levels of serum diamine oxidase, an enzyme that degrades histamine, may play a role in generating headache in gluten sensitivity (23). 

**Fibromyalgia**

Fibromyalgia is a debilitating condition of unknown cause with symptoms that include chronic pain, sleep disturbance, irritability, poor concentration, and fatigue. Symptomatic treatments are only partially effective. Twenty patients with this condition, in whom a diagnosis of CD was excluded by negative anti-tissue transglutaminase antibody results and no villous atrophy in duodenal biopsies, were given a GFD (24). In all patients, improvement in pain was dramatic, and in 15 it was resolved. In three who had attended pain units, opioids could be stopped. Fifteen individuals returned to work or a normal life. Improvement occurred in some patients within a few months but in others was more protracted. In eight cases, ingesting gluten resulted in a worsening of symptoms that subsided on returning to a strict GFD. The same research group followed a series of 246 patients with fibromyalgia, and 90 had experienced improvement on a GFD of the order already observed. A case report supported the link between NCGS and fibromyalgia and the beneficial effects of a GFD (25).

**Low back pain**

A hypothesis was advanced that NCGS may be associated with chronic low back pain related to spondyloarthritis which may be alleviated by a GFD (26, 27). One hundred and ten subjects with severe chronic refractory low back pain suspected of spondyloarthritis who had been on a GFD for at least 4 months were recruited to a study (27). All had negative CD specific serological tests (anti-transglutaminase and anti-deamidated gliadin peptide) before commencing the GFD, and of 89 small bowel biopsies available, none showed villous atrophy. Overall, 69 (62%) experienced significant improvement in low back pain based on criteria such as resolution of symptoms, return to work or normal life, moving from bed to wheelchair, and discontinuation or reduction of analgesics on GFD. Of these 69 patients, 56 began ingesting gluten again and 54 (96%) experienced a worsening of symptoms. Eighteen (16%) and 23 (21%) patients reported partial and no improvement, respectively. It was concluded that more than half of the 110 cases could be classified as having NCGS, as they showed clear improvement on a GFD and deterioration after taking gluten again. This dietary treatment of low backache and the avoidance of analgesics merits further consideration. 

**Reproductive system**

The frequency of gynaecological symptoms and recurrent cystitis was evaluated in 68 women with NCWS diagnosed by a double-blind placebo-controlled wheat challenge (28). Control groups were formed of 71 healthy people, 56 with CD and 52 with irritable bowel syndrome. A structured questionnaire was employed to collect information. Those reporting problems were seen by specialists. 

Most of those with NCWS (59%) admitted having gynaecological symptoms, which is significantly more than for the other groups. Menstrual irregularities were significantly more common in patients than in the controls, and patients suffered significantly more from recurrent vaginitis and dyspareunia. Recurrent cystitis was significantly more frequent in the NCWS group (29%) than in the other groups. Of interest, recurrent vaginitis and cystitis were not associated consistently with infection, and this is still to be explained. On a wheat-free diet for twelve months, menstrual problems resolved in 46% and recurrent vaginitis in 36% of cases. Further evaluation of gynaecological disorders by means of a wheat-free diet is warranted. 

**Cutaneous disorders**

Dermatitis was present in 18% of patients with a presumptive diagnosis of NCGS (11). Seventeen patients with NCGS diagnosed by double-blind placebo-controlled gluten challenge were identified who had skin rashes resembling psoriasis and eczema that benefitted from a GFD and disappeared after a mean duration on the diet of one month, much shorter than for dermatitis herpetiformis (29).

## Conclusions

A brief account of co-morbidities associated with NCGS is presented. The list of conditions, already impressive, will undoubtedly lengthen as time goes by. Awareness that wheat sensitivity might account for unexplained symptoms in some individuals without CD or wheat allergy that may resolve on a GFD is important if optimum care is to be provided for a growing cohort of patients.

## Conflict of interests

The authors declare that they have no conflict of interest.
